# Nonlinear regulation enhances the phenotypic expression of *trans-*acting genetic polymorphisms

**DOI:** 10.1186/1752-0509-1-32

**Published:** 2007-07-25

**Authors:** Arne B Gjuvsland, Ben J Hayes, Theo HE Meuwissen, Erik Plahte, Stig W Omholt

**Affiliations:** 1 Centre for Integrative Genetics (CIGENE), Norwegian University of Life Sciences, Ås, Norway; 2 Department of Animal and Aquacultural Sciences, Norwegian University of Life Sciences, Ås, Norway; 3 Department of Chemistry, Biotechnology, and Food Science, Norwegian University of Life Sciences, Ås, Norway; 4 Animal Genetics and Genomics, Department of Primary Industries, Attwood, Victoria, Australia

## Abstract

**Background:**

Genetic variation explains a considerable part of observed phenotypic variation in gene expression networks. This variation has been shown to be located both locally (*cis*) and distally (*trans*) to the genes being measured. Here we explore to which degree the phenotypic manifestation of local and distant polymorphisms is a dynamic feature of regulatory design.

**Results:**

By combining mathematical models of gene expression networks with genetic maps and linkage analysis we find that very different network structures and regulatory motifs give similar *cis*/*trans *linkage patterns. However, when the shape of the *cis-*regulatory input functions is more nonlinear or threshold-like, we observe for all networks a dramatic increase in the phenotypic expression of distant compared to local polymorphisms under otherwise equal conditions.

**Conclusion:**

Our findings indicate that genetic variation affecting the form of *cis*-regulatory input functions may reshape the genotype-phenotype map by changing the relative importance of *cis *and *trans *variation. Our approach combining nonlinear dynamic models with statistical genetics opens up for a systematic investigation of how functional genetic variation is translated into phenotypic variation under various systemic conditions.

## Background

The key disciplinary goal of genetics the last 100 years has been to understand the relationship between genetic variation and phenotypic variation. A series of concepts have been conceived to describe various aspects of the genotype-phenotype map. Many of them reflect the fact that the phenotypic signatures of alleles and genes depend on other alleles and genes (as for example "dominance" [1] and "epistasis" [2]). However, these concepts have to be regarded as descriptory rather than explanatory. An explanatory theory capable of linking genetic variation with phenotypic variation in even simple mechanistic terms has yet to emerge [3]. However, there exist a few well studied model systems such as the lambda switch [4,5] where this link has been described very well.

An empirically sound starting point for such a theory development will be the mRNA phenotype. The genotype-phenotype gap is in this case narrow compared to higher level phenotypes and relatively simple dynamic models can be used to describe much of the systemic behaviour [6-9]. Also, numerous studies have established that a significant fraction of observed inter-individual variability in gene expression is due to *cis*-linked and *trans*-linked genetic polymorphisms (reviewed by [10] and [11]). How biological systems translate genetic variation into phenotypic variation has recently received some attention [12-16], but there is still an almost completely unrevealed relationship between regulatory polymorphisms, network design principles, and descriptory concepts like *cis*/*trans- *linkage, dominance, epistasis and penetrance even at the expression level.

Gjuvsland *et al*. [17] showed that gene regulatory networks generate significant amounts of *statistical epistasis *which depends on the type of feedback regulation involved. Here we address how single gene descriptors and their dependence on (genetically controlled) regulatory design features contribute to the *functional epistasis *characteristics of mathematical genotype-phenotype maps. Functional epistasis is here used as a common term for describing situations where the phenotypic effect of a genetic substitution (on one or multiple loci) depends on the genetic background, i.e. on the state of other loci in the genotype [18].

The basic strategy underlying our analysis was to (i) position a fixed number of genes on a genetic map; (ii) introduce dynamic network models for the expression of these genes; (iii) define alleles by a set of model parameters and the equilibrium concentrations of the gene products (with noise added) as these genes' expression phenotype; (iv) introduce genetic variation in the model parameters; (v) make mapping populations of individuals having their expression phenotypes determined by the dynamic network models; and (vi) analyse the populations with the machinery of statistical genetics. This approach opens for a systematic investigation of the phenotypic manifestations of genetic variation as a function of gene network design.

As the steady state abundance of mRNA is dependent on the balance between synthesis and decay, our models include one term for synthesis and one for decay of mRNA.

A polymorphism that has an effect on expression level of a given gene *x *must transmit this effect through the production and/or degradation term describing the time rate of change of expression of *x*. This low-resolution modelling approach catches the most important aggregate features of more detailed first-principle models of transcription based on statistical mechanics [19-21] Moreover, the current resolution of empirical data on the existence of non-coding polymorphisms affecting maximal production rates [22-25] and decay rates [26,27] does not invite to make use of more detailed models of the processes underlying these observations. Thus, by letting the parameters defining production rate and decay rate mediate genetic variation in our genotype-phenotype models we account for a whole range of different, and possibly still unrevealed, mechanistic processes responsible for this variation.

**Figure 1:**
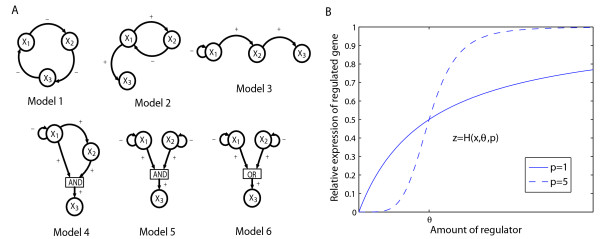
**Interaction diagrams and *cis*-regulatory input function**. (**A**) Interaction diagrams describing the six gene regulatory networks used in the simulations. A circle represents a gene, a +(-)-signed arrow from gene A to gene B symbolises that the gene product of A activates (inhibits) B. The six networks described in regulatory terms are: Model 1: a negative feedback loop with three genes; Model 2: a negative feedback loop with two genes and downstream activation; Model 3: a regulatory chain of three genes; Model 4: a coherent feedforward loop; Model 5: a double input module with AND gate, and Model 6: a double input module with OR gate. (**B**) The Hill function, used as the *cis-*regulatory input function in the models, plotted with constant threshold for various Hill coefficients.

More specifically, we constructed six different three-gene regulatory models (see Methods) based on the transcriptional regulatory motifs that have been characterized in *E. coli *[28] and *S. cerevisiae *[29]: a negative feedback loop with all three genes, a negative feedback loop with two genes and downstream activation, a regulatory chain of three genes, a coherent feedforward loop, a double input module with an AND gate, and a double input module with an OR gate (Figure 1A). For each model we generated genetic variation by sampling maximal production rates, decay rates and regulation thresholds from uniform distributions and assigning them to alleles. In dynamic models of specific biological systems the relevant phenotypes are normally given by some aspect of the solution of the model in a quite straight-forward way. Welch et al [16] introduce a phenotype functional, which transform a dynamic solution in form of a function or time series into a real-valued phenotype, they exemplify such functionals for plant phenotypes; grainfill is represented by cumulative effects, while budding is modelled by threshold triggers. Another example is a model of the lambda switch [4,5] where the two stable steady states of the system correspond to lytic and lysogenic growth phenotypes respectively. However, in a study like the present one where one searches for common characteristics over a whole range of various models, it is not so obvious how to define a common phenotype as different regulatory systems have different properties. A simple property shared by many networks is the stable steady state, and this property is closely associated with homeostatic regulation, which is an ubiquitous property of biological systems [30,31]. For simplicity, we have thus restricted ourselves to systems and parameter values that give a unique stable equilibrium. The stable concentrations of all three gene products for a given set of parameter values (i.e. the genotype) were taken as the genotypic values for the three expression phenotypes (see Methods).

We particularly investigated whether or not different gene network structures create different *cis*- and *trans- *linkage patterns, and how the manifestation of phenotypic effects is influenced by the actual form of the *cis*-regulatory input function [32]. This function (also called gene regulation function [25]) describes the relationship between the production rates of a given gene product and the concentrations of the regulatory agents controlling these rates. Our motivation for focusing on this function is thus that it is both a basic regulatory design common to all network structure and the prime mediator of *trans*-acting effects, in both downstream and feedback regulatory relationships [33]. We chose to work with two distinct functional shapes (or modes), one describing ordinary hyperbolic saturation kinetics (being close to linear over much of the concentration span), and one describing moderately nonlinear (sigmoidal) saturation kinetics (Figure 1B). There is solid empirical [25,34-36] as well as theoretical [19-21,37-39] support for frequent presence of both modes in eukaryotes, and experimental studies have shown that it is relatively easy in mutational terms to move between a hyperbolic mode and a sigmoidal one [25,34]. We found that the shape of the cis-regulatory input function has a dramatic influence on the genotype-phenotype map concerning the phenotypic expression of distant compared to local polymorphisms under otherwise equal conditions.

## Results and Discussion

**Figure 2:**
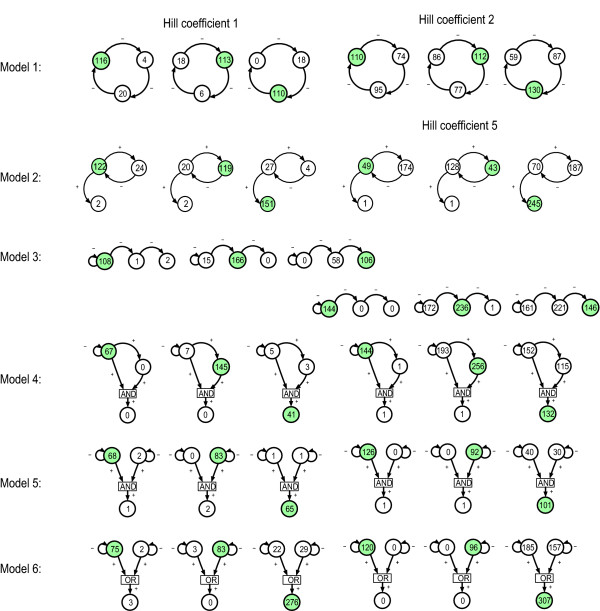
**Linkage analysis on simulated expression phenotypes**. Results from Haley-Knott regression for the six models in Figure 1A. In all plots the gene whose expression QTLs are mapped, is represented with a filled circle. The number inside a circle indicates the number of F2 datasets of the total 400 in which the gene is detected as a QTL for the trait being studied. For instance the leftmost plot of model 1, with *p *= 1, shows that when mapping QTLs for the expression level of gene 1, we detected gene 1 itself in 116 of 400 F2 populations, gene 2 and gene 3 were detected in 4 and 20 F2 populations, respectively. Some false positives are seen when a QTL is reported in the position of a gene downstream of the gene whose expression is being studied.

In all six models the transition from a hyperbolic to a sigmoidal *cis*-regulatory input function causes a dramatic increase in the frequency of detected *trans*-acting determinants (Figure 2). This applied to genes having their gene products explicitly incorporated in the regulatory function of a down-stream gene that is measured as well as those that mediate their regulatory effect through another gene. Further, in five of six models (2, 3, 4, 5, 6) the number of *cis*-linked polymorphisms increases also substantially for at least one of the three mapping instances, but the change is not as dramatic as in the *trans *case. These two patterns are thus quite generic. Despite that polymorphisms in a double input module with an AND gate seem much less prone to be detected than those with an OR gate, the results suggest that even very different gene network structures do not in general cause markedly different *cis*- and *trans-*linkage patterns. Generally, results from multiple trait mappings were very similar to those from the single trait analysis except that fewer false positives were detected (results not shown).

**Figure 3:**
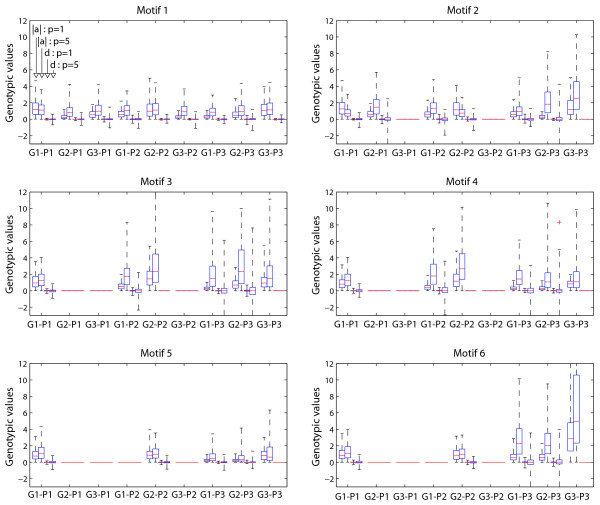
**Additive and dominance genotypic values**. Box plots for all six models of the additive and dominance genotypic values *a *and *d *of all three genes on the expression phenotypes of all the three genes. For each model the plots are organized into four and four boxes named "G *i *- P *j *" indicating that the genotypic values of gene *i *for the expression level of gene *j *is plotted. The four box plots in each group show the distribution across the 400 F2 populations of  |*a*|, *p*=1,  |*a*|, *p*=5,  *d*, *p*=1,  *d*, *p*=5 (for model 1 p = 2 was used instead of 5), respectively.

How can these observations be interpreted in biological terms? Linkage analysis estimates so-called additive (*a*) and dominance (*d*) genotypic values (see Methods). For a single locus with two alleles *a *measures half the distance between the genotypic values of the two homozygots. In other words, this parameter describes the mean difference between the two homozygote genotypes in phenotypic units. The dominance genotypic value is the difference between the heterozygot genotyic value and the midpoint of the two homozygots. The gene action of the locus is described by these two parameters; if *d *= 0 the locus is said to be additive, if *d *< | *a *| it shows partial dominance, complete dominance if *d *< | *a *| and overdominance if *d *< | *a *|. Together, *a *and *d *constitute the basic gene action model upon which the predictive machinery of quantitative genetics, which includes variance components, heritabilities and breeding values, is built (see [40]). These two values thus constitute the link between the mathematical genotype-phenotype models and the linkage analysis results. When we compare the distributions of additive and dominance values in the hyperbolic and sigmoidal case for all the models, the general pattern is that the additive and dominance absolute values become more fanned out for local as well as for distant variation when the steepness of the *cis-*regulatory input function increases (Figure 3). This means that steepening the *cis*-regulatory input function causes in general a given set of regulatory polymorphisms to have more distinct phenotypic signatures. In accordance with the linkage analysis results (Figure 2), the steepening modulates the allelic variation such that the phenotypic expression effect of distant polymorphisms increases relative to the local polymorphisms. We also see that in the hyperbolic case, dominance effects are scarcely present, while in the sigmoidal case they contribute substantially. As the change of shape of the *cis*-regulatory input function can be interpreted as a change of genetic background, the observed shifts in the *cis-/trans*-linkage and dominance patterns are manifestations of functional epistasis in the actual genotype-phenotype map.

**Figure 4:**
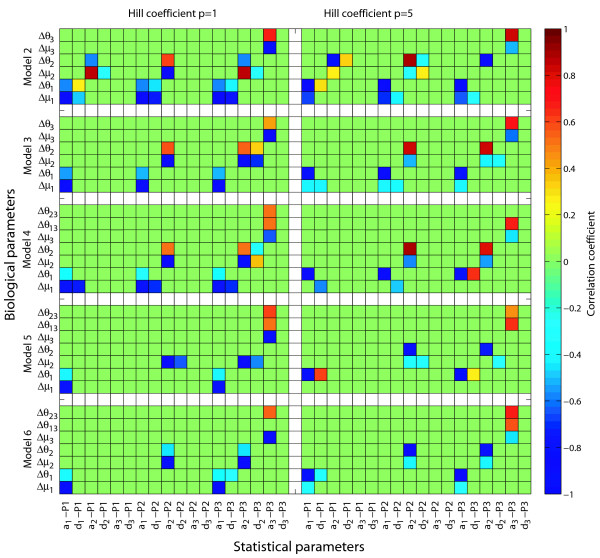
**Dynamic model parameters and genotypic values**. Correlation coefficients between genotypic values and allelic differences in values of biological parameters, in datasets of 400 simulated F2 populations for model 2 through 6 (where the highest Hill coefficient p = 5 is used). Correlations that are weaker than a threshold determined by permutations (see Methods) are set to zero. On the horizontal axis " a_*i *_- P_*j*_" and " d_*i *_- P_*j*_" denotes the genotypic values of gene *i *for the expression level of gene *j*. On the vertical axis parameter value differences between allele 1 and 2 (from parental lines P1 and P2 respectively) of gene *i *are denoted by Δμi=μi1−μi2=αi1γi1−αi2γi2
 MathType@MTEF@5@5@+=feaafiart1ev1aqatCvAUfKttLearuWrP9MDH5MBPbIqV92AaeXatLxBI9gBaebbnrfifHhDYfgasaacH8akY=wiFfYdH8Gipec8Eeeu0xXdbba9frFj0=OqFfea0dXdd9vqai=hGuQ8kuc9pgc9s8qqaq=dirpe0xb9q8qiLsFr0=vr0=vr0dc8meaabaqaciaacaGaaeqabaqabeGadaaakeaacqGHuoariiGacqWF8oqBdaWgaaWcbaGaemyAaKgabeaakiabg2da9iab=X7aTnaaBaaaleaacqWGPbqAcqaIXaqmaeqaaOGaeyOeI0Iae8hVd02aaSbaaSqaaiabdMgaPjabikdaYaqabaGccqGH9aqpdaWcaaqaaiab=f7aHnaaBaaaleaacqWGPbqAcqaIXaqmaeqaaaGcbaGae83SdC2aaSbaaSqaaiabdMgaPjabigdaXaqabaaaaOGaeyOeI0YaaSaaaeaacqWFXoqydaWgaaWcbaGaemyAaKMaeGOmaidabeaaaOqaaiab=n7aNnaaBaaaleaacqWGPbqAcqaIYaGmaeqaaaaaaaa@4E44@ and threshold value Δ*θ*_*i *_= *θ*_*i*1 _- *θ*_*i*2 _(see Methods for further explanation for these parameters).

The linkage analysis results (Figure 2) can thus be explained by how allelic effects on *a *and *d *values are systemically modulated. However, this elucidation does not reveal the actual relationships between genetic variation in production rates, decay rates and regulation thresholds and the phenotypic *a *and *d *values. Intuitively one would expect that polymorphisms affecting maximal production rates or decay rates would appear as eQTLs. For instance, Schadt et al [41] highlights polymorphisms affecting the decay rate of C5, and a double copy number variation of Alad, which will increase the production capacity, as candidate polymorphisms for *cis*-acting eQTLs in mice. The relationship between polymorphisms affecting the shape of the *cis*-regulatory input function is less intuitive. With mathematical models of gene regulatory networks we can explore these relationships in detail. Some very interesting features emerge from a systematic investigation of how allelic differences in these three parameters for a given gene are correlated with its own expression as well as the expression of all other genes in the network in terms of *a *and *d *(Figure 4, results for models 2 through 6 where the steepest *cis*-regulatory input function was used). The correlation between additive genotypic values and parental line difference (see legend to Figure 4) in the ratio between maximal production rate and relative decay rate (*μ*) and the threshold value (*θ*)changes dramatically with the steepness of the *cis*-regulatory input function. This is true for additive genotypic values underlying both *cis- *and *trans*-linkage. Furthermore, the effect of going from the hyperbolic case to the sigmoidal case is that differences in *μ *becomes more weakly correlated with *a*, while the differences in *θ *become stronger correlated to *a*. In molecular terms this suggests that mutations affecting the steepness of the *cis*-regulatory input function will alter the phenotypic effect of other polymorphisms, simultaneously releasing variation associated with one type of parameter and buffering variation associated with the other. Other simulation studies have also shown that both maintenance and release of genetic variation are emergent properties of gene regulatory networks with sigmoidal dose-response relationships [42,43], and our findings identify the steepness as an important modulator of genetic variation. Another aspect is that if *trans*- and *cis*-linked polymorphisms are both selected for in a given network this implicitly leads to more pronounced dependency patterns compared to when only *cis*-linked polymorphisms are selected for. The frequency of *trans-*linked polymorphisms identified by linkage analysis [11] strongly suggest that selection for such polymorphisms is a frequent phenomenon.

With regard to dominance values, all models contain cases where these are correlated with *μ *or *θ *or both (Figure 4). However, the relationship between model parameters and dominance values is dramatically weaker than for additive values. This implies that *cis*-regulatory variation at a given locus does not relate in a straightforward manner to dominance values associated with its own gene product or on gene products of down-stream loci. Predictors of the dominance variation can be constructed in deterministic models like the ones made use of here, but these predictors will have to include much more extensive information about the system. The variation in dominance effects is thus a more pronounced systemic feature than the variation in additive effects, which in turn implies that the classical definition of dominance as an intralocus interaction [40,44] should be used with care.

Although *cis*-regulatory variation is more difficult to detect and understand [45,46], we have focused on the phenotypic signatures of *cis*-regulatory variation in transcriptional networks and not taken into account the effect of coding polymorphisms. The rationale for this is that genetical genomics studies in yeast [47] and mice [48] strongly suggest that this *cis-*variation is a very important cause for self-linkage.

Our results apply to a much wider range of regulatory settings than what appears from a superficial inspection of the differential equations (see Methods). This is because a regulatory relationship can be mediated by numerous other gene products influencing a variety of intra- and intercellular processes, a simple example being a transcriptional cascade [35]. As long as all these gene products simply transfer the signal between genes A and B in the form of a well-defined dose-response functional relationship, the complexity of this transduction does not influence our predictions. Sigmoidal gene regulation functions are widely used in models of gene networks. Here we employ the frequently [6,9,14,49] used Hill function (see Methods). Properties of the transcriptional machinery such as multiple transcription factor binding sites, synergy and cooperativity [37], and fractal kinetics [38] will contribute to sigmoidal gene regulation functions. Mathematical description of transcription regulation by use of statistical mechanics methodology [19-21] as well as experimental data [25,35,36] also suggest that the Hill function is very well suited for describing a whole range of mechanistic processes causing nonlinear transcription responses.

Simulations with genotype-phenotype maps defined by genotypic values is a widely used tool in quantitative genetics, and the main purposes are demonstrating and testing methods for mapping of QTLs [50-52]. Such simulations are very useful for showing differences between various mapping methods, and for identifying weaknesses of current methodologies. The main difference to the approach presented here is that we start out from a dynamic system of genes rather than statistical effects. The genotypic values, which are explicitly defined in the genetic model approach, instead become emergent properties of a biologically interpretable dynamic system. This opens up for a much deeper understanding of functional epistasis aspects [18] of the genotype-phenotype map in terms of biological processes and mechanisms. This is illustrated by our identification of the *cis-*regulatory input function as an important provider of functional epistasis to the genotype-phenotype map, which is clearly beyond reach for the standard genetic model approach.

In the case of sigmoidal gene regulation functions, our results (Figure 4) indicate that polymorphisms affecting *μ *(the ratio between maximal production rate and relative decay rate) will not be directly translated into a QTL effect on the steady state. This opens a new opportunity window for genetical genomics studies. Although frequently considered to be the phenotype level closest to DNA sequence variation [11], transcript abundance does actually reflect the balance between production and decay rates. These two rates are thus more directly tied up with DNA sequence variation than transcript abundance. Genome-wide studies of decay rates have already been performed in yeast [53] and human cells [54], and in principle such data could be used to map rateQTLs in the same way as they are used for expressionQTLs. As our results illustrate how variation can be visible at one phenotypic level and hidden at the next level for systemic reasons, comparing QTLs for rates and expression levels can thus probably be exploited to reveal to which degree systemic silencing of mutations in transcriptional networks is a generic feature or not.

## Conclusion

When mathematical models capable of bridging the genotype-phenotype gap are embedded in a framework accounting for the number of individuals, mating structures, allele frequencies, genome-wide variations in recombination frequencies and linkage disequilibrium structures, we possess a tool to understand how various polymorphisms affect phenotypic variation in a population. With our simple models we have here only sketched the potential of this approach, but the methodis likely to be applicable also in more complex settings. Although we in this paper focus on expression networks, there is in principle no limit to how many systemic levels one can include, and how sophisticated the mathematical phenotypes can be [55]. Our approach thus opens up for a systematic investigation of the systemic conditions under which different types of functional genetic variation make detectable contributions to the phenotypic variation of traits of interest to biomedicine, production biology and evolutionary biology. The main constraint will be our capacity to make biologically realistic mathematical descriptions of complex phenotypes over a broad range, not the structural complexities of the genetic variation involved.

## Methods

### Gene regulatory model equations

For modelling gene regulatory networks we use the sigmoid formalism [56,57] for diploid organisms [14]. A gene regulatory network is described by a set of ordinary differential equations (ODEs):

dx¯dt=F(x¯,α¯,γ¯,θ¯,p),
 MathType@MTEF@5@5@+=feaafiart1ev1aaatCvAUfKttLearuWrP9MDH5MBPbIqV92AaeXatLxBI9gBaebbnrfifHhDYfgasaacH8akY=wiFfYdH8Gipec8Eeeu0xXdbba9frFj0=OqFfea0dXdd9vqai=hGuQ8kuc9pgc9s8qqaq=dirpe0xb9q8qiLsFr0=vr0=vr0dc8meaabaqaciaacaGaaeqabaqabeGadaaakeaadaWcaaqaaiabdsgaKjqbdIha4zaaraaabaGaemizaqMaemiDaqhaaiabg2da9iabdAeagjabcIcaOiqbdIha4zaaraGaeiilaWccciGaf8xSdeMbaebacqGGSaalcuWFZoWzgaqeaiabcYcaSiqb=H7aXzaaraGaeiilaWIaemiCaaNaeiykaKIaeiilaWcaaa@42C8@

where the 2n-vector x¯
 MathType@MTEF@5@5@+=feaafiart1ev1aqatCvAUfKttLearuWrP9MDH5MBPbIqV92AaeXatLxBI9gBaebbnrfifHhDYfgasaacH8akY=wiFfYdH8Gipec8Eeeu0xXdbba9frFj0=OqFfea0dXdd9vqai=hGuQ8kuc9pgc9s8qqaq=dirpe0xb9q8qiLsFr0=vr0=vr0dc8meaabaqaciaacaGaaeqabaqabeGadaaakeaacuWG4baEgaqeaaaa@2E3E@ = (*x*_11 _*x*_12 _*x*_21 _*x*_22 _... x_*n*1 _x_*n*2_) contains the expression levels *x*_*i*1_, *x*_*i*2 _of the products of the two alleles for gene number *i*, *i *= 1, 2, ..., *n*, in the gene regulatory network, the vectors α¯
 MathType@MTEF@5@5@+=feaafiart1ev1aqatCvAUfKttLearuWrP9MDH5MBPbIqV92AaeXatLxBI9gBaebbnrfifHhDYfgasaacH8akY=wiFfYdH8Gipec8Eeeu0xXdbba9frFj0=OqFfea0dXdd9vqai=hGuQ8kuc9pgc9s8qqaq=dirpe0xb9q8qiLsFr0=vr0=vr0dc8meaabaqaciaacaGaaeqabaqabeGadaaakeaaiiGacuWFXoqygaqeaaaa@2E6B@, γ¯
 MathType@MTEF@5@5@+=feaafiart1ev1aqatCvAUfKttLearuWrP9MDH5MBPbIqV92AaeXatLxBI9gBaebbnrfifHhDYfgasaacH8akY=wiFfYdH8Gipec8Eeeu0xXdbba9frFj0=OqFfea0dXdd9vqai=hGuQ8kuc9pgc9s8qqaq=dirpe0xb9q8qiLsFr0=vr0=vr0dc8meaabaqaciaacaGaaeqabaqabeGadaaakeaaiiGacuWFZoWzgaqeaaaa@2E73@ and θ¯
 MathType@MTEF@5@5@+=feaafiart1ev1aqatCvAUfKttLearuWrP9MDH5MBPbIqV92AaeXatLxBI9gBaebbnrfifHhDYfgasaacH8akY=wiFfYdH8Gipec8Eeeu0xXdbba9frFj0=OqFfea0dXdd9vqai=hGuQ8kuc9pgc9s8qqaq=dirpe0xb9q8qiLsFr0=vr0=vr0dc8meaabaqaciaacaGaaeqabaqabeGadaaakeaaiiGacuWF4oqCgaqeaaaa@2E82@ contain allelic parameter values, and *p *determines the steepness of the *cis*-regulatory input function (see below). To each allele, we associate the parameters *a*_*ij*_, the maximal production rate of the allele, and *γ*_*ij*_, the relative decay rate of the expression product. In addition, for each gene *x*_*k*. _regulating the expression of *x*_*ij*_, there is a threshold parameter, *θ*_*kij *_used to describe the dose-response relationship, or the regulatory function, between *x*_*k*. _and the resulting production rate of *x*_*ij*_. We assumed that the two allele products are equally efficient as regulators so their levels are summed (*y*_*i *_= *x*_*i*1 _+ *x*_*i*2_) before they are used in the regulatory function. The Hill function [58] generates a flexible dose-response relationship between regulator and production at the regulated gene:

H(y,θ,p)=ypθp+yp,
 MathType@MTEF@5@5@+=feaafiart1ev1aqatCvAUfKttLearuWrP9MDH5MBPbIqV92AaeXatLxBI9gBaebbnrfifHhDYfgasaacH8akY=wiFfYdH8Gipec8Eeeu0xXdbba9frFj0=OqFfea0dXdd9vqai=hGuQ8kuc9pgc9s8qqaq=dirpe0xb9q8qiLsFr0=vr0=vr0dc8meaabaqaciaacaGaaeqabaqabeGadaaakeaacqWGibascqGGOaakcqWG5bqEcqGGSaaliiGacqWF4oqCcqGGSaalcqWGWbaCcqGGPaqkcqGH9aqpdaWcaaqaaiabdMha5naaCaaaleqabaGaemiCaahaaaGcbaGae8hUde3aaWbaaSqabeaacqWGWbaCaaGccqGHRaWkcqWG5bqEdaahaaWcbeqaaiabdchaWbaaaaGccqGGSaalaaa@4238@

where *θ *gives the amount of regulator needed to get 50% of maximal production rate while *p *determines the steepness of the response. The Hill equation describes Michaelis-Menten regulation for *p *= 1 and switchlike response as *p *increases (Figure 1B). We varied *p *between simulations, but within replicates of a particular scenario *p *is fixed both between alleles and across regulatory actions. If the regulatory effect is inhibitory, the regulatory function 1 - *H*(*y*,*θ*,*p*) is used.

Six diploid mathematical models of the interaction diagrams in Figure 1A were made using the sigmoid formalism. In all the equations *j *= 1,2 and *y*_*j *_= *x*_*j*1 _+ *x*_*j*2_, *i *= 1,2,3.

Model 1: Negative feedback loop with 3 genes

x˙1j=α1j(1−H(y3,θ31j,p))−γ1jx1j,x˙2j=α2j(1−H(y1,θ12j,p))−γ2jx2j,x˙3j=α3j(1−H(y2,θ23j,p))−γ3jx3j.
 MathType@MTEF@5@5@+=feaafiart1ev1aaatCvAUfKttLearuWrP9MDH5MBPbIqV92AaeXatLxBI9gBaebbnrfifHhDYfgasaacH8akY=wiFfYdH8Gipec8Eeeu0xXdbba9frFj0=OqFfea0dXdd9vqai=hGuQ8kuc9pgc9s8qqaq=dirpe0xb9q8qiLsFr0=vr0=vr0dc8meaabaqaciaacaGaaeqabaqabeGadaaakqaaeeqaaiqbdIha4zaacaWaaSbaaSqaaiabigdaXiabdQgaQbqabaGccqGH9aqpiiGacqWFXoqydaWgaaWcbaGaeGymaeJaemOAaOgabeaakiabcIcaOiabigdaXiabgkHiTiabdIeaijabcIcaOiabdMha5naaBaaaleaacqaIZaWmaeqaaOGaeiilaWIae8hUde3aaSbaaSqaaiabiodaZiabigdaXiabdQgaQbqabaGccqGGSaalcqWGWbaCcqGGPaqkcqGGPaqkcqGHsislcqWFZoWzdaWgaaWcbaGaeGymaeJaemOAaOgabeaakiabdIha4naaBaaaleaacqaIXaqmcqWGQbGAaeqaaOGaeiilaWcabaGafmiEaGNbaiaadaWgaaWcbaGaeGOmaiJaemOAaOgabeaakiabg2da9iab=f7aHnaaBaaaleaacqaIYaGmcqWGQbGAaeqaaOGaeiikaGIaeGymaeJaeyOeI0IaemisaGKaeiikaGIaemyEaK3aaSbaaSqaaiabigdaXaqabaGccqGGSaalcqWF4oqCdaWgaaWcbaGaeGymaeJaeGOmaiJaemOAaOgabeaakiabcYcaSiabdchaWjabcMcaPiabcMcaPiabgkHiTiab=n7aNnaaBaaaleaacqaIYaGmcqWGQbGAaeqaaOGaemiEaG3aaSbaaSqaaiabikdaYiabdQgaQbqabaGccqGGSaalaeaacuWG4baEgaGaamaaBaaaleaacqaIZaWmcqWGQbGAaeqaaOGaeyypa0Jae8xSde2aaSbaaSqaaiabiodaZiabdQgaQbqabaGccqGGOaakcqaIXaqmcqGHsislcqWGibascqGGOaakcqWG5bqEdaWgaaWcbaGaeGOmaidabeaakiabcYcaSiab=H7aXnaaBaaaleaacqaIYaGmcqaIZaWmcqWGQbGAaeqaaOGaeiilaWIaemiCaaNaeiykaKIaeiykaKIaeyOeI0Iae83SdC2aaSbaaSqaaiabiodaZiabdQgaQbqabaGccqWG4baEdaWgaaWcbaGaeG4mamJaemOAaOgabeaakiabc6caUaaaaa@9A0B@

Model 2: Negative feedback loop with 2 genes, downstream activation

x˙1j=α1j(1−H(y2,θ21j,p))−γ1jx1j,x˙2j=α2jH(y1,θ12j,p)−γ2jx2j,x˙3j=α3jH(y1,θ13j,p)−γ3jx3j.
 MathType@MTEF@5@5@+=feaafiart1ev1aaatCvAUfKttLearuWrP9MDH5MBPbIqV92AaeXatLxBI9gBaebbnrfifHhDYfgasaacH8akY=wiFfYdH8Gipec8Eeeu0xXdbba9frFj0=OqFfea0dXdd9vqai=hGuQ8kuc9pgc9s8qqaq=dirpe0xb9q8qiLsFr0=vr0=vr0dc8meaabaqaciaacaGaaeqabaqabeGadaaakqaaeeqaaiqbdIha4zaacaWaaSbaaSqaaiabigdaXiabdQgaQbqabaGccqGH9aqpiiGacqWFXoqydaWgaaWcbaGaeGymaeJaemOAaOgabeaakiabcIcaOiabigdaXiabgkHiTiabdIeaijabcIcaOiabdMha5naaBaaaleaacqaIYaGmaeqaaOGaeiilaWIae8hUde3aaSbaaSqaaiabikdaYiabigdaXiabdQgaQbqabaGccqGGSaalcqWGWbaCcqGGPaqkcqGGPaqkcqGHsislcqWFZoWzdaWgaaWcbaGaeGymaeJaemOAaOgabeaakiabdIha4naaBaaaleaacqaIXaqmcqWGQbGAaeqaaOGaeiilaWcabaGafmiEaGNbaiaadaWgaaWcbaGaeGOmaiJaemOAaOgabeaakiabg2da9iab=f7aHnaaBaaaleaacqaIYaGmcqWGQbGAaeqaaOGaemisaGKaeiikaGIaemyEaK3aaSbaaSqaaiabigdaXaqabaGccqGGSaalcqWF4oqCdaWgaaWcbaGaeGymaeJaeGOmaiJaemOAaOgabeaakiabcYcaSiabdchaWjabcMcaPiabgkHiTiab=n7aNnaaBaaaleaacqaIYaGmcqWGQbGAaeqaaOGaemiEaG3aaSbaaSqaaiabikdaYiabdQgaQbqabaGccqGGSaalaeaacuWG4baEgaGaamaaBaaaleaacqaIZaWmcqWGQbGAaeqaaOGaeyypa0Jae8xSde2aaSbaaSqaaiabiodaZiabdQgaQbqabaGccqWGibascqGGOaakcqWG5bqEdaWgaaWcbaGaeGymaedabeaakiabcYcaSiab=H7aXnaaBaaaleaacqaIXaqmcqaIZaWmcqWGQbGAaeqaaOGaeiilaWIaemiCaaNaeiykaKIaeyOeI0Iae83SdC2aaSbaaSqaaiabiodaZiabdQgaQbqabaGccqWG4baEdaWgaaWcbaGaeG4mamJaemOAaOgabeaakiabc6caUaaaaa@92E5@

Model 3: Regulatory chain with 3 genes

x˙1j=α1j(1−H(y1,θ11j,p))−γ1jx1j,x˙2j=α2jH(y1,θ12j,p)−γ2jx2j,x˙3j=α3jH(y2,θ23j,p)−γ3jx3j.
 MathType@MTEF@5@5@+=feaafiart1ev1aaatCvAUfKttLearuWrP9MDH5MBPbIqV92AaeXatLxBI9gBaebbnrfifHhDYfgasaacH8akY=wiFfYdH8Gipec8Eeeu0xXdbba9frFj0=OqFfea0dXdd9vqai=hGuQ8kuc9pgc9s8qqaq=dirpe0xb9q8qiLsFr0=vr0=vr0dc8meaabaqaciaacaGaaeqabaqabeGadaaakqaaeeqaaiqbdIha4zaacaWaaSbaaSqaaiabigdaXiabdQgaQbqabaGccqGH9aqpiiGacqWFXoqydaWgaaWcbaGaeGymaeJaemOAaOgabeaakiabcIcaOiabigdaXiabgkHiTiabdIeaijabcIcaOiabdMha5naaBaaaleaacqaIXaqmaeqaaOGaeiilaWIae8hUde3aaSbaaSqaaiabigdaXiabigdaXiabdQgaQbqabaGccqGGSaalcqWGWbaCcqGGPaqkcqGGPaqkcqGHsislcqWFZoWzdaWgaaWcbaGaeGymaeJaemOAaOgabeaakiabdIha4naaBaaaleaacqaIXaqmcqWGQbGAaeqaaOGaeiilaWcabaGafmiEaGNbaiaadaWgaaWcbaGaeGOmaiJaemOAaOgabeaakiabg2da9iab=f7aHnaaBaaaleaacqaIYaGmcqWGQbGAaeqaaOGaemisaGKaeiikaGIaemyEaK3aaSbaaSqaaiabigdaXaqabaGccqGGSaalcqWF4oqCdaWgaaWcbaGaeGymaeJaeGOmaiJaemOAaOgabeaakiabcYcaSiabdchaWjabcMcaPiabgkHiTiab=n7aNnaaBaaaleaacqaIYaGmcqWGQbGAaeqaaOGaemiEaG3aaSbaaSqaaiabikdaYiabdQgaQbqabaGccqGGSaalaeaacuWG4baEgaGaamaaBaaaleaacqaIZaWmcqWGQbGAaeqaaOGaeyypa0Jae8xSde2aaSbaaSqaaiabiodaZiabdQgaQbqabaGccqWGibascqGGOaakcqWG5bqEdaWgaaWcbaGaeGOmaidabeaakiabcYcaSiab=H7aXnaaBaaaleaacqaIYaGmcqaIZaWmcqWGQbGAaeqaaOGaeiilaWIaemiCaaNaeiykaKIaeyOeI0Iae83SdC2aaSbaaSqaaiabiodaZiabdQgaQbqabaGccqWG4baEdaWgaaWcbaGaeG4mamJaemOAaOgabeaakiabc6caUaaaaa@92E5@

For the last three models with regulatory functions involving double inputs, the following logical functions were used:

AND(Z1,Z2)=Z1Z2,OR(Z1,Z2)=Z1+Z2−Z1Z2.
 MathType@MTEF@5@5@+=feaafiart1ev1aaatCvAUfKttLearuWrP9MDH5MBPbIqV92AaeXatLxBI9gBaebbnrfifHhDYfgasaacH8akY=wiFfYdH8Gipec8Eeeu0xXdbba9frFj0=OqFfea0dXdd9vqai=hGuQ8kuc9pgc9s8qqaq=dirpe0xb9q8qiLsFr0=vr0=vr0dc8meaabaqaciaacaGaaeqabaqabeGadaaakqaabeqaaGqaaiab=feabjab=5eaojab=reaejabcIcaOiabdQfaAnaaBaaaleaacqaIXaqmaeqaaOGaeiilaWIaemOwaO1aaSbaaSqaaiabikdaYaqabaGccqGGPaqkcqGH9aqpcqWGAbGwdaWgaaWcbaGaeGymaedabeaakiabdQfaAnaaBaaaleaacqaIYaGmaeqaaOGaeiilaWcabaGae83ta8Kae8NuaiLaeiikaGIaemOwaO1aaSbaaSqaaiabigdaXaqabaGccqGGSaalcqWGAbGwdaWgaaWcbaGaeGOmaidabeaakiabcMcaPiabg2da9iabdQfaAnaaBaaaleaacqaIXaqmaeqaaOGaey4kaSIaemOwaO1aaSbaaSqaaiabikdaYaqabaGccqGHsislcqWGAbGwdaWgaaWcbaGaeGymaedabeaakiabdQfaAnaaBaaaleaacqaIYaGmaeqaaOGaeiOla4caaaa@54E8@

Model 4: Coherent feedforward loop

x˙1j=α1j(1−H(y1,θ11j,p))−γ1jx1j,x˙2j=α2jH(y1,θ12j,p)−γ2jx2j,x˙3j=α3jAND(H(y1,θ13j,p),H(y2,θ23j,p))−γ3jx3j.
 MathType@MTEF@5@5@+=feaafiart1ev1aaatCvAUfKttLearuWrP9MDH5MBPbIqV92AaeXatLxBI9gBaebbnrfifHhDYfgasaacH8akY=wiFfYdH8Gipec8Eeeu0xXdbba9frFj0=OqFfea0dXdd9vqai=hGuQ8kuc9pgc9s8qqaq=dirpe0xb9q8qiLsFr0=vr0=vr0dc8meaabaqaciaacaGaaeqabaqabeGadaaakqaaeeqaaiqbdIha4zaacaWaaSbaaSqaaiabigdaXiabdQgaQbqabaGccqGH9aqpiiGacqWFXoqydaWgaaWcbaGaeGymaeJaemOAaOgabeaakiabcIcaOiabigdaXiabgkHiTiabdIeaijabcIcaOiabdMha5naaBaaaleaacqaIXaqmaeqaaOGaeiilaWIae8hUde3aaSbaaSqaaiabigdaXiabigdaXiabdQgaQbqabaGccqGGSaalcqWGWbaCcqGGPaqkcqGGPaqkcqGHsislcqWFZoWzdaWgaaWcbaGaeGymaeJaemOAaOgabeaakiabdIha4naaBaaaleaacqaIXaqmcqWGQbGAaeqaaOGaeiilaWcabaGafmiEaGNbaiaadaWgaaWcbaGaeGOmaiJaemOAaOgabeaakiabg2da9iab=f7aHnaaBaaaleaacqaIYaGmcqWGQbGAaeqaaOGaemisaGKaeiikaGIaemyEaK3aaSbaaSqaaiabigdaXaqabaGccqGGSaalcqWF4oqCdaWgaaWcbaGaeGymaeJaeGOmaiJaemOAaOgabeaakiabcYcaSiabdchaWjabcMcaPiabgkHiTiab=n7aNnaaBaaaleaacqaIYaGmcqWGQbGAaeqaaOGaemiEaG3aaSbaaSqaaiabikdaYiabdQgaQbqabaGccqGGSaalaeaacuWG4baEgaGaamaaBaaaleaacqaIZaWmcqWGQbGAaeqaaOGaeyypa0Jae8xSde2aaSbaaSqaaiabiodaZiabdQgaQbqabaacbaGccqGFbbqqcqGFobGtcqGFebarcqGGOaakcqWGibascqGGOaakcqWG5bqEdaWgaaWcbaGaeGymaedabeaakiabcYcaSiab=H7aXnaaBaaaleaacqaIXaqmcqaIZaWmcqWGQbGAaeqaaOGaeiilaWIaemiCaaNaeiykaKIaeiilaWIaemisaGKaeiikaGIaemyEaK3aaSbaaSqaaiabikdaYaqabaGccqGGSaalcqWF4oqCdaWgaaWcbaGaeGOmaiJaeG4mamJaemOAaOgabeaakiabcYcaSiabdchaWjabcMcaPiabcMcaPiabgkHiTiab=n7aNnaaBaaaleaacqaIZaWmcqWGQbGAaeqaaOGaemiEaG3aaSbaaSqaaiabiodaZiabdQgaQbqabaGccqGGUaGlaaaa@A66F@

Model 5: Double input, AND block

x˙1j=α1j(1−H(y1,θ11j,p))−γ1jx1j,x˙2j=α2j(1−H(y2,θ22j,p))−γ2jx2j,x˙3j=α3jAND(H(y1,θ13j,p),H(y2,θ23j,p))−γ3jx3j.
 MathType@MTEF@5@5@+=feaafiart1ev1aaatCvAUfKttLearuWrP9MDH5MBPbIqV92AaeXatLxBI9gBaebbnrfifHhDYfgasaacH8akY=wiFfYdH8Gipec8Eeeu0xXdbba9frFj0=OqFfea0dXdd9vqai=hGuQ8kuc9pgc9s8qqaq=dirpe0xb9q8qiLsFr0=vr0=vr0dc8meaabaqaciaacaGaaeqabaqabeGadaaakqaaeeqaaiqbdIha4zaacaWaaSbaaSqaaiabigdaXiabdQgaQbqabaGccqGH9aqpiiGacqWFXoqydaWgaaWcbaGaeGymaeJaemOAaOgabeaakiabcIcaOiabigdaXiabgkHiTiabdIeaijabcIcaOiabdMha5naaBaaaleaacqaIXaqmaeqaaOGaeiilaWIae8hUde3aaSbaaSqaaiabigdaXiabigdaXiabdQgaQbqabaGccqGGSaalcqWGWbaCcqGGPaqkcqGGPaqkcqGHsislcqWFZoWzdaWgaaWcbaGaeGymaeJaemOAaOgabeaakiabdIha4naaBaaaleaacqaIXaqmcqWGQbGAaeqaaOGaeiilaWcabaGafmiEaGNbaiaadaWgaaWcbaGaeGOmaiJaemOAaOgabeaakiabg2da9iab=f7aHnaaBaaaleaacqaIYaGmcqWGQbGAaeqaaOGaeiikaGIaeGymaeJaeyOeI0IaemisaGKaeiikaGIaemyEaK3aaSbaaSqaaiabikdaYaqabaGccqGGSaalcqWF4oqCdaWgaaWcbaGaeGOmaiJaeGOmaiJaemOAaOgabeaakiabcYcaSiabdchaWjabcMcaPiabcMcaPiabgkHiTiab=n7aNnaaBaaaleaacqaIYaGmcqWGQbGAaeqaaOGaemiEaG3aaSbaaSqaaiabikdaYiabdQgaQbqabaGccqGGSaalaeaacuWG4baEgaGaamaaBaaaleaacqaIZaWmcqWGQbGAaeqaaOGaeyypa0Jae8xSde2aaSbaaSqaaiabiodaZiabdQgaQbqabaacbaGccqGFbbqqcqGFobGtcqGFebarcqGGOaakcqWGibascqGGOaakcqWG5bqEdaWgaaWcbaGaeGymaedabeaakiabcYcaSiab=H7aXnaaBaaaleaacqaIXaqmcqaIZaWmcqWGQbGAaeqaaOGaeiilaWIaemiCaaNaeiykaKIaeiilaWIaemisaGKaeiikaGIaemyEaK3aaSbaaSqaaiabikdaYaqabaGccqGGSaalcqWF4oqCdaWgaaWcbaGaeGOmaiJaeG4mamJaemOAaOgabeaakiabcYcaSiabdchaWjabcMcaPiabcMcaPiabgkHiTiab=n7aNnaaBaaaleaacqaIZaWmcqWGQbGAaeqaaOGaemiEaG3aaSbaaSqaaiabiodaZiabdQgaQbqabaGccqGGUaGlaaaa@AA02@

Model 6: Double input, OR block

x˙1j=α1j(1−H(y1,θ11j,p))−γ1jx1i,x˙2j=α2j(1−H(y2,θ22j,p))−γ2jx2i,x˙3j=α3jOR(H(y1,θ13j,p),H(y2,θ23j,p))−γ3jx3i.
 MathType@MTEF@5@5@+=feaafiart1ev1aaatCvAUfKttLearuWrP9MDH5MBPbIqV92AaeXatLxBI9gBaebbnrfifHhDYfgasaacH8akY=wiFfYdH8Gipec8Eeeu0xXdbba9frFj0=OqFfea0dXdd9vqai=hGuQ8kuc9pgc9s8qqaq=dirpe0xb9q8qiLsFr0=vr0=vr0dc8meaabaqaciaacaGaaeqabaqabeGadaaakqaaeeqaaiqbdIha4zaacaWaaSbaaSqaaiabigdaXiabdQgaQbqabaGccqGH9aqpiiGacqWFXoqydaWgaaWcbaGaeGymaeJaemOAaOgabeaakiabcIcaOiabigdaXiabgkHiTiabdIeaijabcIcaOiabdMha5naaBaaaleaacqaIXaqmaeqaaOGaeiilaWIae8hUde3aaSbaaSqaaiabigdaXiabigdaXiabdQgaQbqabaGccqGGSaalcqWGWbaCcqGGPaqkcqGGPaqkcqGHsislcqWFZoWzdaWgaaWcbaGaeGymaeJaemOAaOgabeaakiabdIha4naaBaaaleaacqaIXaqmcqWGPbqAaeqaaOGaeiilaWcabaGafmiEaGNbaiaadaWgaaWcbaGaeGOmaiJaemOAaOgabeaakiabg2da9iab=f7aHnaaBaaaleaacqaIYaGmcqWGQbGAaeqaaOGaeiikaGIaeGymaeJaeyOeI0IaemisaGKaeiikaGIaemyEaK3aaSbaaSqaaiabikdaYaqabaGccqGGSaalcqWF4oqCdaWgaaWcbaGaeGOmaiJaeGOmaiJaemOAaOgabeaakiabcYcaSiabdchaWjabcMcaPiabcMcaPiabgkHiTiab=n7aNnaaBaaaleaacqaIYaGmcqWGQbGAaeqaaOGaemiEaG3aaSbaaSqaaiabikdaYiabdMgaPbqabaGccqGGSaalaeaacuWG4baEgaGaamaaBaaaleaacqaIZaWmcqWGQbGAaeqaaOGaeyypa0Jae8xSde2aaSbaaSqaaiabiodaZiabdQgaQbqabaacbaGccqGFpbWtcqGFsbGucqGGOaakcqWGibascqGGOaakcqWG5bqEdaWgaaWcbaGaeGymaedabeaakiabcYcaSiab=H7aXnaaBaaaleaacqaIXaqmcqaIZaWmcqWGQbGAaeqaaOGaeiilaWIaemiCaaNaeiykaKIaeiilaWIaemisaGKaeiikaGIaemyEaK3aaSbaaSqaaiabikdaYaqabaGccqGGSaalcqWF4oqCdaWgaaWcbaGaeGOmaiJaeG4mamJaemOAaOgabeaakiabcYcaSiabdchaWjabcMcaPiabcMcaPiabgkHiTiab=n7aNnaaBaaaleaacqaIZaWmcqWGQbGAaeqaaOGaemiEaG3aaSbaaSqaaiabiodaZiabdMgaPbqabaGccqGGUaGlaaaa@A914@

### Genetic map

The same genetic map was used for all simulations. This map contained five 100 cM chromosomes, and marker loci were spaced equidistant at each 5 cM along the chromosomes. The three genes were placed at the three first chromosomes, gene 1 at c1-42.5 cM, gene 2 at c2-22.5 cM and gene 3 at c3-57.5 cM. Haldane's mapping function was used to compute recombination rates between loci.

### Simulations

For each of the six gene regulatory network models two simulations were run with different Hill coefficients in the regulatory functions. Hill coefficients 1 and 5 were used for models 2–6, but for model 1 Hill coefficient 5 gave cyclic behaviour, and the steepness was reduced to Hill coefficient 2. We started by sampling allelic parameter values from independent uniform distribution of all three types of heritable model parameters, maximal production rate *α*, threshold for regulation *θ*, and relative decay rate *γ*, such that 70 ≤ *α *≤ 150, 5 ≤ *θ *≤ 15, and 10 ≤ *γ *≤ 15. To allele *i *of gene *j *we associated *α*_*ij*_, *γ*_*ij *_and one or two *θ*_*kij *_depending on the model and gene. For a given diploid genotype consisting of parameter values for two alleles at each of the three genes the resulting system of equations was solved to find the stable equilibrium values for all three genes, and these simulated expression levels were used as the genotype's contribution to the phenotype. To get the individual phenotype record, independent normally distributed noise with mean 0 and variance 25 was added to the genotypic contribution. For each network model and Hill coefficient we created a set of mapping populations by sampling allelic parameter values for 40 fully homozygous lines, half (P1-lines) of these lines were homozygous 11 at all marker loci, the other half (P2-lines) were homozygous 22. Finally, each P1-line was crossed to each P2-line in an F2-cross. For each gene regulatory model and Hill coefficient this gave 400 F2 populations for the genetic analysis.

### Linkage analysis

Haley-Knott regression was done using the function scanone in the R\qtl package [59], while Multitrait IM was done with the function JZmapqtl in QTL Cartographer [60,61]. Genome wide 5% significance thresholds for LOD and LR scores were set by applying both methods to 2500 F2 populations of 250 individuals, using the same genetic map as in the simulations, but with only environmental noise contributing to the expression levels. For both methods the test statistic was computed every 2.5 cM along the whole genetic map. If a test statistic exceeded the threshold a QTL was inferred, however, at most one QTL was flagged from each chromosome.

### Evaluation of QTL results

By comparing the positions of the three genes underlying the simulated phenotypes to flagged QTL positions, genes were divided into two groups: detected and not detected. A gene was classified as detected if a significant QTL was flagged on the chromosome at which the gene resided and the QTL peak was in the same marker bracket as the gene or in one of the neighbour brackets (i.e. ≤ 7.5 cM away from the gene), otherwise the gene was classified as not detected.

### Genotypic values

The concept of *value*, expressible in the metric units of the phenotype is central in quantitative genetics. The phenotype observed in an individual is the *phenotypic value *of that individual and this is divided into components attributable to influence of the genotype, the *genotypic value*, and the environment [40]. The genotypic value of a genotype is the mean phenotypic value of individuals with that genotype. In our simulated data genotypic values were calculated before adding noise to the steady state expression levels. Following the notation used by [44,62] and extending to three genes *G*_*ijklmn *_denotes the genotypic value of an individual with genotype *ij *at gene 1, *kl *at gene 2, and *mn *at gene 3, where *ij*,*kl*,*mn *= 11,12,22. Single locus genotypic values are defined by the unweighted average of the 9 genotypic values across the other loci,

Gij....=Gij1111+Gij1211+Gij2211+Gij1112+Gij1212+Gij2212+Gij1122+Gij1222+Gij22229,
 MathType@MTEF@5@5@+=feaafiart1ev1aaatCvAUfKttLearuWrP9MDH5MBPbIqV92AaeXatLxBI9gBaebbnrfifHhDYfgasaacH8akY=wiFfYdH8Gipec8Eeeu0xXdbba9frFj0=OqFfea0dXdd9vqai=hGuQ8kuc9pgc9s8qqaq=dirpe0xb9q8qiLsFr0=vr0=vr0dc8meaabaqaciaacaGaaeqabaqabeGadaaakeaacqWGhbWrdaWgaaWcbaGaemyAaKMaemOAaOMaeiOla4IaeiOla4IaeiOla4IaeiOla4cabeaakiabg2da9maalaaabaGaem4raC0aaSbaaSqaaiabdMgaPjabdQgaQjabigdaXiabigdaXiabigdaXiabigdaXaqabaGccqGHRaWkcqWGhbWrdaWgaaWcbaGaemyAaKMaemOAaOMaeGymaeJaeGOmaiJaeGymaeJaeGymaedabeaakiabgUcaRiabdEeahnaaBaaaleaacqWGPbqAcqWGQbGAcqaIYaGmcqaIYaGmcqaIXaqmcqaIXaqmaeqaaOGaey4kaSIaem4raC0aaSbaaSqaaiabdMgaPjabdQgaQjabigdaXiabigdaXiabigdaXiabikdaYaqabaGccqGHRaWkcqWGhbWrdaWgaaWcbaGaemyAaKMaemOAaOMaeGymaeJaeGOmaiJaeGymaeJaeGOmaidabeaakiabgUcaRiabdEeahnaaBaaaleaacqWGPbqAcqWGQbGAcqaIYaGmcqaIYaGmcqaIXaqmcqaIYaGmaeqaaOGaey4kaSIaem4raC0aaSbaaSqaaiabdMgaPjabdQgaQjabigdaXiabigdaXiabikdaYiabikdaYaqabaGccqGHRaWkcqWGhbWrdaWgaaWcbaGaemyAaKMaemOAaOMaeGymaeJaeGOmaiJaeGOmaiJaeGOmaidabeaakiabgUcaRiabdEeahnaaBaaaleaacqWGPbqAcqWGQbGAcqaIYaGmcqaIYaGmcqaIYaGmcqaIYaGmaeqaaaGcbaGaeGyoaKdaaiabcYcaSaaa@8458@

at gene 1,

G..kl..=G11kl11+G12kl11+G22kl11+G11kl12+G12kl12+G22kl12+G11kl22+G12kl22+G22kl229,
 MathType@MTEF@5@5@+=feaafiart1ev1aaatCvAUfKttLearuWrP9MDH5MBPbIqV92AaeXatLxBI9gBaebbnrfifHhDYfgasaacH8akY=wiFfYdH8Gipec8Eeeu0xXdbba9frFj0=OqFfea0dXdd9vqai=hGuQ8kuc9pgc9s8qqaq=dirpe0xb9q8qiLsFr0=vr0=vr0dc8meaabaqaciaacaGaaeqabaqabeGadaaakeaacqWGhbWrdaWgaaWcbaGaeiOla4IaeiOla4Iaem4AaSMaemiBaWMaeiOla4IaeiOla4cabeaakiabg2da9maalaaabaGaem4raC0aaSbaaSqaaiabigdaXiabigdaXiabdUgaRjabdYgaSjabigdaXiabigdaXaqabaGccqGHRaWkcqWGhbWrdaWgaaWcbaGaeGymaeJaeGOmaiJaem4AaSMaemiBaWMaeGymaeJaeGymaedabeaakiabgUcaRiabdEeahnaaBaaaleaacqaIYaGmcqaIYaGmcqWGRbWAcqWGSbaBcqaIXaqmcqaIXaqmaeqaaOGaey4kaSIaem4raC0aaSbaaSqaaiabigdaXiabigdaXiabdUgaRjabdYgaSjabigdaXiabikdaYaqabaGccqGHRaWkcqWGhbWrdaWgaaWcbaGaeGymaeJaeGOmaiJaem4AaSMaemiBaWMaeGymaeJaeGOmaidabeaakiabgUcaRiabdEeahnaaBaaaleaacqaIYaGmcqaIYaGmcqWGRbWAcqWGSbaBcqaIXaqmcqaIYaGmaeqaaOGaey4kaSIaem4raC0aaSbaaSqaaiabigdaXiabigdaXiabdUgaRjabdYgaSjabikdaYiabikdaYaqabaGccqGHRaWkcqWGhbWrdaWgaaWcbaGaeGymaeJaeGOmaiJaem4AaSMaemiBaWMaeGOmaiJaeGOmaidabeaakiabgUcaRiabdEeahnaaBaaaleaacqaIYaGmcqaIYaGmcqWGRbWAcqWGSbaBcqaIYaGmcqaIYaGmaeqaaaGcbaGaeGyoaKdaaiabcYcaSaaa@84A8@

at gene 2, and

G....mn=G1111mn+G1211mn+G2211mn+G1112mn+G1212mn+G2212mn+G1122mn+G1222mn+G2222mn9
 MathType@MTEF@5@5@+=feaafiart1ev1aaatCvAUfKttLearuWrP9MDH5MBPbIqV92AaeXatLxBI9gBaebbnrfifHhDYfgasaacH8akY=wiFfYdH8Gipec8Eeeu0xXdbba9frFj0=OqFfea0dXdd9vqai=hGuQ8kuc9pgc9s8qqaq=dirpe0xb9q8qiLsFr0=vr0=vr0dc8meaabaqaciaacaGaaeqabaqabeGadaaakeaacqWGhbWrdaWgaaWcbaGaeiOla4IaeiOla4IaeiOla4IaeiOla4IaemyBa0MaemOBa4gabeaakiabg2da9maalaaabaGaem4raC0aaSbaaSqaaiabigdaXiabigdaXiabigdaXiabigdaXiabd2gaTjabd6gaUbqabaGccqGHRaWkcqWGhbWrdaWgaaWcbaGaeGymaeJaeGOmaiJaeGymaeJaeGymaeJaemyBa0MaemOBa4gabeaakiabgUcaRiabdEeahnaaBaaaleaacqaIYaGmcqaIYaGmcqaIXaqmcqaIXaqmcqWGTbqBcqWGUbGBaeqaaOGaey4kaSIaem4raC0aaSbaaSqaaiabigdaXiabigdaXiabigdaXiabikdaYiabd2gaTjabd6gaUbqabaGccqGHRaWkcqWGhbWrdaWgaaWcbaGaeGymaeJaeGOmaiJaeGymaeJaeGOmaiJaemyBa0MaemOBa4gabeaakiabgUcaRiabdEeahnaaBaaaleaacqaIYaGmcqaIYaGmcqaIXaqmcqaIYaGmcqWGTbqBcqWGUbGBaeqaaOGaey4kaSIaem4raC0aaSbaaSqaaiabigdaXiabigdaXiabikdaYiabikdaYiabd2gaTjabd6gaUbqabaGccqGHRaWkcqWGhbWrdaWgaaWcbaGaeGymaeJaeGOmaiJaeGOmaiJaeGOmaiJaemyBa0MaemOBa4gabeaakiabgUcaRiabdEeahnaaBaaaleaacqaIYaGmcqaIYaGmcqaIYaGmcqaIYaGmcqWGTbqBcqWGUbGBaeqaaaGcbaGaeGyoaKdaaaaa@8418@

at gene 3. The additive genotypic value is half the distance between the homozygote genotypic values, while the dominance genotypic value is the deviation of the heterozygote genotypic value from the midpoint of the homozygote genotypic value. Using gene 1 as an example we get,

a1=G11...−G22....2,
 MathType@MTEF@5@5@+=feaafiart1ev1aaatCvAUfKttLearuWrP9MDH5MBPbIqV92AaeXatLxBI9gBaebbnrfifHhDYfgasaacH8akY=wiFfYdH8Gipec8Eeeu0xXdbba9frFj0=OqFfea0dXdd9vqai=hGuQ8kuc9pgc9s8qqaq=dirpe0xb9q8qiLsFr0=vr0=vr0dc8meaabaqaciaacaGaaeqabaqabeGadaaakeaacqWGHbqydaWgaaWcbaGaeGymaedabeaakiabg2da9maalaaabaGaem4raC0aaSbaaSqaaiabigdaXiabigdaXiabc6caUiabc6caUiabc6caUaqabaGccqGHsislcqWGhbWrdaWgaaWcbaGaeGOmaiJaeGOmaiJaeiOla4IaeiOla4IaeiOla4IaeiOla4cabeaaaOqaaiabikdaYaaacqGGSaalaaa@3F8C@

and

d1=G12....−G11...+G22....2.
 MathType@MTEF@5@5@+=feaafiart1ev1aaatCvAUfKttLearuWrP9MDH5MBPbIqV92AaeXatLxBI9gBaebbnrfifHhDYfgasaacH8akY=wiFfYdH8Gipec8Eeeu0xXdbba9frFj0=OqFfea0dXdd9vqai=hGuQ8kuc9pgc9s8qqaq=dirpe0xb9q8qiLsFr0=vr0=vr0dc8meaabaqaciaacaGaaeqabaqabeGadaaakeaacqWGKbazdaWgaaWcbaGaeGymaedabeaakiabg2da9iabdEeahnaaBaaaleaacqaIXaqmcqaIYaGmcqGGUaGlcqGGUaGlcqGGUaGlcqGGUaGlaeqaaOGaeyOeI0YaaSaaaeaacqWGhbWrdaWgaaWcbaGaeGymaeJaeGymaeJaeiOla4IaeiOla4IaeiOla4cabeaakiabgUcaRiabdEeahnaaBaaaleaacqaIYaGmcqaIYaGmcqGGUaGlcqGGUaGlcqGGUaGlcqGGUaGlaeqaaaGcbaGaeGOmaidaaiabc6caUaaa@4737@

### Correlation coefficients

For each model and Hill coefficient, vectors of genotypic values and allelic differences in values of network model parameters were collected. These vectors had 400 elements, each representing one F2 population. Correlation coefficients for all pair-wise combinations of statistical and network model parameters were computed. The significance of each of these correlation coefficients was evaluated by computing the correlation coefficients of 1000 permutations, reshuffling elements at random within one of the vectors. Correlation coefficients falling inside the interval observed in the permuted datasets were set to zero.

## Authors' contributions

ABG was responsible for the design of the study, the modelling, simulations, statistical analysis and for drafting the manuscript. BJH contributed to the simulation work, statistical analysis and drafting of the manuscript. THEM contributed to the statistical analysis. EP contributed to design of models and simulations. SWO conceived the research and contributed to the modelling, simulations, statistical analysis and writing of the manuscript. All authors read and approved the final manuscript.
